# Current understanding of the *Trypanosoma cruzi*-cardiomyocyte interaction

**DOI:** 10.3389/fimmu.2012.00327

**Published:** 2012-10-30

**Authors:** Claudia M. Calvet, Tatiana G. Melo, Luciana R. Garzoni, Francisco O. R. Oliveira Jr., Dayse T. Silva Neto, Maria N. S. L., L. Meirelles, Mirian C. S. Pereira

**Affiliations:** Laboratório de Ultra-estrutura Celular, Fundação Oswaldo Cruz, Instituto Oswaldo CruzRio de Janeiro, Rio de Janeiro, Brazil

**Keywords:** *Trypanosoma cruzi*, cardiomyocyte, cell recognition, endocytosis, cytoskeleton, cell junction, extracellular matrix, apoptosis

## Abstract

*Trypanosoma cruzi*, the etiological agent of Chagas disease, exhibits multiple strategies to ensure its establishment and persistence in the host. Although this parasite has the ability to infect different organs, heart impairment is the most frequent clinical manifestation of the disease. Advances in knowledge of *T. cruzi*–cardiomyocyte interactions have contributed to a better understanding of the biological events involved in the pathogenesis of Chagas disease. This brief review focuses on the current understanding of molecules involved in *T. cruzi*–cardiomyocyte recognition, the mechanism of invasion, and on the effect of intracellular development of *T. cruzi* on the structural organization and molecular response of the target cell.

## INTRODUCTION

Chagas disease, caused by *Trypanosoma cruzi* infection, has emerged as an important global public health problem due to the many Latin American *T. cruzi*-infected immigrants in non-endemic countries (Pérez-Molina et al., 2012). Although public health programs in the Southern Cone countries have reduced transmission by 70% (Moncayo and Silveira, 2009), blood and organ transplant transmissions in non-endemic countries (Rassi et al., 2009) and outbreaks of foodborne transmission (Pereira et al., 2009; Ríos et al., 2011) have drawn attention to Chagas disease. An estimated 8–15 million individuals in 18 endemic countries in Central and South America are infected, with approximately 30 million people at risk (WHO, 2010; Rassi et al., 2012). Chronic chagasic cardiomyopathy, the most relevant clinical manifestation, is the leading cause of death from heart failure in endemic countries, and accounts for a significant burden of ischemic and inflammatory heart disease in the USA and Europe due to “globalization” of Chagas disease (Moncayo and Silveira, 2009; Moolani et al., 2012). In this review, we summarize current knowledge of the biology of the *T. cruzi*–host cell interaction, highlighting molecular aspects of *T. cruzi*–cardiomyocyte interplay, with a focus on early infection events and the effect of intracellular parasite development on the structure and function of the target cell.

## CELL RECOGNITION AND INVASION PROCESS

### *T. cruzi*–CARDIOMYOCYTE RECOGNITION

**FIGURE 1:**
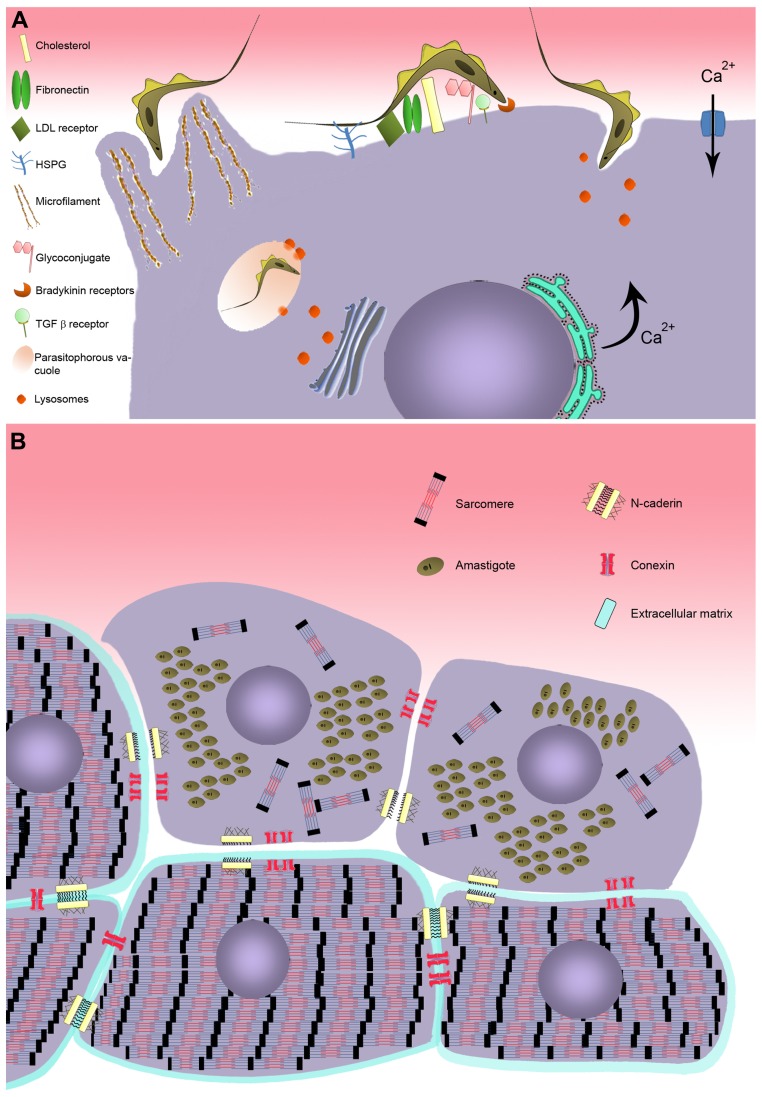
**Model of *T. cruzi* invasion in cardiomyocyte. (A)** Schematic model representing the recognition step involving cell surface molecules of cardiomyocyte during *T. cruzi* invasion. Two distinct mechanism of invasion are represented: actin-dependent and lysosome-dependent mechanisms. **(B)** Effect of *T. cruzi* infection on cardiomyocyte structure. Disturbance on the cardiomyocyte cytoarchitecture is evidenced after *T. cruzi* infection, showing breakdown of myofibrillar and disruption of adherent and gap junctions.

Interplay between parasite and host cell is essential for *T. cruzi* to successfully adjust to the different microenvironments it occupies in its vertebrate and invertebrate hosts. In the obligatory intracellular phase of its life cycle in the mammalian host, infection is driven by adhesion and internalization events involving a large variety of ligands and/or receptors on the surface of both the parasite and host cell interacting with one another to achieve recognition and invasion. Several different surface molecules in the cardiomyocyte have been implicated in adhesion and internalization by the parasite (**Figure 1**). Carbohydrate residues of membrane glycoconjugates in cardiomyocytes, including galactosyl, mannosyl, and sialyl residues, participate in *T. cruzi* cytoadherence (Barbosa and Meirelles, 1992, 1993), while mannose receptors at the surface of cardiomyocytes modulate parasite entry and are down-regulated by *T. cruzi* infection (Soeiro et al., 1999).

Extracellular matrix (ECM) components are also important in parasite–host cell recognition. Fibronectin, a high molecular weight glycoprotein present at the host cell surface, is recognized by fibronectin receptors of the parasite (Ouaissi et al., 1984), which interact with the RGDS (Arg-Gly-Asp-Ser) sequence of fibronectin and mediate parasite entry (Calvet et al., 2004). Immunization with RGDS peptide induced protection in an experimental murine model of acute *T. cruzi* infection (Ouaissi et al., 1986). Heparan sulfate proteoglycans (HSPG), another class of ECM component widely distributed in mammalian tissues, are also involved in *T. cruzi* attachment and invasion (Ortega-Barria and Pereira, 1991; Calvet et al., 2003). Treatment of trypomastigotes and amastigotes, the infective forms of *T. cruzi*, or cardiomyocytes with soluble heparan sulfate (HS) and heparitinase II, respectively, efficiently inhibited parasite invasion (Calvet et al., 2003; Oliveira Jr. et al., 2008; Bambino-Medeiros et al., 2011). The binding of *T. cruzi* to HSPG involves the recognition of the *N*-acetylated/*N*-sulfated domain of the HS chain by heparin-binding proteins (HBPs) present at the surface of the parasite (Oliveira Jr. et al., 2008). Although *T. cruzi* HBPs are capable of binding HS and chondroitin sulfate (CS), only the HS–HBPs interaction triggers parasite invasion in cardiomyocytes (Calvet et al., 2003; Oliveira Jr. et al., 2008), while HS and CS are involved in vector–*T. cruzi* interactions (Oliveira Jr. et al., 2012).

Lipids also play an important role in *T. cruzi*–host cell interplay. Membrane rafts, enriched in cholesterol and sphingolipids, appear to participate in the invasion process (Barrias et al., 2007; Fernandes et al., 2007; Priotto et al., 2009). Recently, cholesterol has been demonstrated to modulate invasion of cardiomyocytes by *T. cruzi* (Hissa et al., 2012). Depletion of cholesterol from cardiac cell membrane induced an 85–90% reduction of parasite invasion by inhibiting parasites’ association with lysosomes. Additionally, the low-density lipoprotein receptor, which is up-regulated in myocardium of infected mice, also coordinates parasite entry and fusion of the parasitophorous vacuole (PV) with lysosomes (Nagajyothi et al., 2011).

### MECHANISMS OF *T.cruzi* INVASION

The large number of molecules involved in recognition of target cells by *T. cruzi* increases the parasite’s capacity to explore multiple strategies to ensure propagation in the mammalian host. A number of different mechanisms of *T. cruzi* invasion have been described, involving distinct host cell type, parasite genotype, and developmental stage. At least five models of invasion have been elucidated. (i) An actin-dependent mechanism leads to the rearrangement of microfilaments, inducing the host cell membrane to enclose the parasite (Barbosa and Meirelles, 1995; Procópio et al., 1999; Rosestolato et al., 2002; Ferreira et al., 2006). (ii) Lysosome-dependent mechanisms, involving an increase of transient cytosolic Ca^2+^ levels induced by the parasite, generate cortical actin depolymerization and lysosome recruitment to the parasite binding site (Rodríguez et al., 1999; Hissa et al., 2012). (iii) Activated signaling pathways also participate, including tyrosine kinase receptors (TrKA and TrKC; de Melo-Jorge and PereiraPerrin, 2007; Weinkauf et al., 2011) and phosphatidylinositol 3-kinase (PI3-K; Todorov et al., 2000; Chuenkova et al., 2001; Wilkowsky et al., 2001; Vieira et al., 2002; Woolsey et al., 2003), bradykinin receptors (Scharfstein et al., 2000; Todorov et al., 2003), and transforming growth factor β (TGF-β; Ming et al., 1995; Waghabi et al., 2007). (iv) More recently, sphingomyelinase-mediated plasma membrane repair has been proposed to participate in this process (Fernandes et al., 2011; Fernandes and Andrews, 2012), as has (v) the host cell autophagy pathway (Romano et al., 2009, 2012). Finally, the combination of different mechanisms has been described as coordinating the* T. cruzi* invasion process (Butler and Tyler, 2012).

Elevation of transient intracellular Ca^2+^ levels, an invasion-related effect provoked by *T. cruzi* binding to the host cell membrane (**Figure 1**), has also been demonstrated in cardiac cells (Barr et al., 1996; Garzoni et al., 2003). The increase of cytosolic [Ca^2+^] has been reported to be brought about in two different ways: (i) by sarcoplasmic reticulum stores, which are sensitive to leupeptin, suggesting a cortical actin depolymerization and lysosome-dependent mechanism of invasion (Barr et al., 1996), and by (ii) extracellular Ca^2+^ influx through membrane Ca^2+^ channels, which are insensitive to leupeptin (Garzoni et al., 2003). Recently, it has been suggested that Ca^2+^ influx may also occur as a result of lesions on the plasma membrane, suggesting that the membrane repair pathway frequently observed in muscle cells may also be involved in cardiac cell invasion by *T. cruzi* (Fernandes and Andrews, 2012).

Transforming growth factor β, a multifunctional family of proteins that controls a range of biological events in most cells, including proliferation and cellular differentiation (Moustakas et al., 2002), has also been shown to participate in *T. cruzi* invasion of cardiomyocytes (Waghabi et al., 2005). *T. cruzi* directly activates latent TGF-β and modulates TGF-β signaling (Waghabi et al., 2005). Inhibition of *T. cruzi* infection in cardiomyocyte was achieved by blockage of the TGF-β receptor type I (TGFβRI)/Smad2 signaling pathway by SB-431542, a TGF-β signaling inhibitor (Waghabi et al., 2007). Besides impairment of parasite invasion, the inhibitor treatment also reduced *T. cruzi* intracellular multiplication and differentiation. Recently, the therapeutic effectiveness of GW788388, an oral inhibitor of TGF-β signaling, has been demonstrated experimentally in acute phase *T. cruzi* infection, leading to a reduction of parasitemia and mortality, and also preventing cardiac fibrosis (de Oliveira et al., 2012).

Bradykinin receptors (B_2_R/B_1_R) have also been reported to be involved in cardiomyocyte infection by *T. cruzi* (Todorov et al., 2003). This mechanism of invasion is regulated by cooperation between HSPG, kininogen, and cruzipain-1, the major cysteine protease isoform of *T. cruzi*, resulting in the release of kinin. Invasion through the kinin transduction pathway, activated by G protein-coupled bradykinin receptors, induces intracellular Ca^2+^ mobilization from stores in the endoplasmic reticulum (Scharfstein et al., 2000). The B_2_R agonist captopril stimulates the invasion of *T. cruzi* while B_2_R and B_1_R antagonists, present inhibitory effects on cardiomyocytes, suggesting that these receptors interdependently drive invasion of the parasite (Todorov et al., 2003).

As evidenced in other non-professional phagocytic cells (Rosestolato et al., 2002; Ferreira et al., 2006), *T. cruzi* entry is also mediated by an endocytic process in cardiac muscle. A protrusion of cardiomyocyte plasma membrane, orchestrated by cytoskeleton rearrangement, is observed during *T. cruzi*–cardiomyocyte interplay. A dense actin-based membrane skeleton meshwork projects from the sarcolemma and encloses the entering parasite (Barbosa and Meirelles, 1995). This event was drastically inhibited (75%) when cardiac cells were treated with cytochalasin D, an agent that depolymerizes actin filaments, prior to *T. cruzi* infection; no parasite invasion was observed in fixed cardiomyocytes (Barbosa and Meirelles, 1995). Once inside the cells, the parasite is located within a PV that lacks Ca^2+^–Mg^2+^-ATPase, adenylate cyclase, and anionic sites (Meirelles et al., 1986) but has carbohydrate residues such as *N*-acetylglucosamine and *N*-acetylgalactosamine (Barbosa and Meirelles, 1992, 1993). Ultrastructural cytochemistry for the lysosomal enzymes aryl sulfatase and acid phosphatase has revealed the fusion of the parasite-containing vacuole with lysosomes (Meirelles et al., 1987). The acidification of the PV by lysosomal fusion, leading to the activation of TC-TOX and disruption of the PV membrane (Andrews et al., 1990; Hall, 1993), is a prerequisite for the trypomastigote to exit the phagosome, also allowing the parasite to be retained intracellularly and complete its life cycle (Andrade and Andrews, 2004, 2005; Mott and Burleigh, 2008).

## EFFECT OF *T. cruzi* INFECTION IN CARDIOMYOCYTE PHYSIOLOGY

During the *T. cruzi*–cardiomyocyte interaction the parasite gains control of overall host cell gene expression, including expression of genes related to immune response, inflammation, cytoskeletal organization, cell–cell and cell–matrix interactions, apoptosis, cell cycle, and oxidative stress (Goldenberg et al., 2009; Manque et al., 2011). The intense trypanocidal immune response generated in cardiomyocytes in response to infection by *T. cruzi* results in the production of cytokines, chemokines, and nitric oxide that, while essential elements of the defensive reaction in cardiac tissue (Machado et al., 2000, 2008; Manque et al., 2011), can also result in cardiac hypertrophy (Petersen and Burleigh, 2003; Waghabi et al., 2009). Several studies report that *T. cruzi* infection stimulates production of nitric oxide synthase 2, matrix metalloproteinase-2 (MMP-2) and MMP-9 in cardiomyocytes, as well as interleukin-6 (IL-6), IL-1β, tumor necrosis factor-alpha and TGF-β (Petersen and Burleigh, 2003; Petersen et al., 2005; Gutierrez et al., 2008; Waghabi et al., 2009; Nogueira de Melo et al., 2010). Peroxisome proliferator-activated receptor γ is also implicated in regulating the inflammatory process (Hovsepian et al., 2011). Moreover, IL-1β-mediated development of cardiomyocyte hypertrophy is orchestrated by Toll-like receptor 2 (Petersen et al., 2005). Proinflammatory cytokines also modulate production of mitochondrial reactive oxygen species, impairing the efficiency of the respiratory chain (Gupta et al., 2009). Mitochondrial disturbance has been identified as an important effect of chagasic cardiomyopathy (Garg et al., 2003; Báez et al., 2011). Inflammatory mediators have also been reported to regulate Rabs expression (Stein et al., 2003) thereby interfering with host cell trafficking. Down-regulation of Rab GTPase proteins, including the effector molecule of Rab5 (EEA1), Rab7, and Rab11, has been demonstrated in *T. cruzi*-infected cardiomyocytes, and it has been proposed that a delayed endocytic pathway may favor microbicidal activity and increase antigen processing (Batista et al., 2006).

**FIGURE 2:**
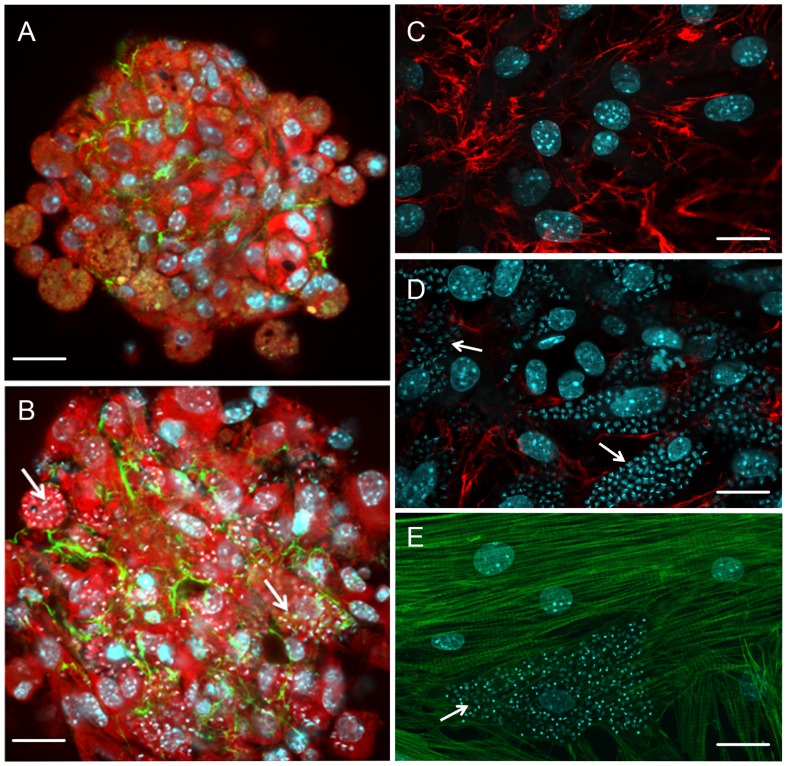
**Effect of *T. cruzi* infection on cardiomyocyte cytoarchitecture and extracellular matrix remodeling (A–E)**. *T. cruzi*-infected cardiomyocyte 3D-culture revealed fibrosis and hypertrophy. Note an increase in spheroid size and the spatial distribution of fibronectin (FN; green) in *T. cruzi*-infected **(B)** compared with the uninfected **(A)** 3D-culture system. However, the FN labeling fainted in highly *T. cruzi*-infected cells. In contrast, the analysis of FN (red) distribution in cardiomyocyte 2D-culture **(C)** demonstrates a reduction of this extracellular matrix component in *T. cruzi*-infected cardiomyocyte culture **(D)**. The low expression of FN in the cells harboring the parasites and the factors involved in the enhancement of FN in 3D-culture are unclear and focus of future investigation. **(E)** Cytoskeletal changes were also evidenced in *T. cruzi*-infected cardiomyocytes, showing complete disorganization of myofibrils. Cardiomyocytes were stained with Evans-blue (red; **A** and **B**) and DAPI (blue; **A–E**), a DNA dye. Arrows indicate intracellular parasites. Bar = 20 µm.

Changes in cytoskeletal proteins have also been shown during parasite intracellular development (**Figures 1 and 2**). The complex cytoskeleton organization of cardiomyocytes involved in the contraction–relaxation process of the heart is affected by *T. cruzi* infection (Pereira et al., 1993; Taniwaki et al., 2006). Breakdown of myofibrils has been seen in areas of amastigote nests (Pereira et al., 1993; Taniwaki et al., 2006) and disturbance of intermediate filaments (desmin) and microtubules was also induced by parasite proliferation (Pereira et al., 1993). Interestingly, the actin isoform mRNAs, α-cardiac and β-actin mRNAs, are altered during the parasite intracellular cycle (Pereira et al., 2000). Down-regulation of α-cardiac actin mRNA concomitant with up-regulation of β-actin mRNA suggested the reactivation of non-differentiated cell program. Also within the context of cytoskeletal changes, actin-binding proteins have been demonstrated to be altered in *T. cruzi*-infected cardiomyocytes. Alpha-actinin, an F-actin crosslinker protein that anchors actin to the Z line, and costameres, repeating adhesion structures consisting of vinculin involved in the lateral transmission of contractility force to the sarcolemma, are disrupted and down-regulated in *T. cruzi*-infected cells, reducing strength and force transduction (Melo et al., 2004, 2006). These cytoskeletal disorders are accompanied by deregulation of Ca^2+^ influx, affecting cardiac cell contractility (Taniwaki et al., 2006). One striking feature of trypanocidal drugs is their effect on the recovery of cardiomyocyte cytoskeleton (Garzoni et al., 2004; Silva et al., 2006; Adesse et al., 2011a). Posaconazole, an ergosterol biosynthesis inhibitor with potent trypanocidal activity currently in clinical trials, has been demonstrated to promote the reassembly of the contractile apparatus and microtubule network in *T. cruzi*-infected cardiomyocytes (Silva et al., 2006). The reorganization of myofibrils leads to recovery of cardiomyocyte functionality. Similarly, treatment of *T. cruzi*-infected cardiomyocyte cultures with bisphosphonate risedronate, a farnesyl pyrophosphate synthase inhibitor, and amiodarona, an anti-arrhythmic drug, also fostered the recovery of myofibrils (Garzoni et al., 2004; Adesse et al., 2011a) and may represent interesting alternatives for Chagas therapy.

In addition to disruption of the cytoskeletal architecture by the parasite, cell–cell adhesion (adherens junctions) and intercellular communication (gap junctions), which play important physiological roles in cardiac tissue, are also been disrupted by *T. cruzi* infection (Adesse et al., 2008, 2011b; Melo et al., 2008). Alteration in spatial distribution and down-regulation of the adherence junction proteins N-cadherin and β-catenin in *T. cruzi*-infected cardiomyocytes (Melo et al., 2008) may interfere with tissue integrity and perturb the function of the cardiac conduction system, as has been proposed to be the case in arrhythmogenic cardiomyopathies (Mezzano and Sheikh, 2012). Additionally, electrical conduction disturbance, frequently seen in both acute and chronic phases of Chagas diseases, seems to be related to altered gap junction (connexin-43) coupling of cardiomyocytes induced by *T. cruzi* (de Carvalho et al., 1992, 1994; Adesse et al., 2008, 2011b). Connexin-43 dysregulation has also been attributed to increased levels of TGF-β (Waghabi et al., 2009). Following treatment of *T. cruzi*-infected cardiomyocyte cultures with amiodarone and SB-431542 causes reversal of the disorganization of gap junctions and return to their normal distribution (Waghabi et al., 2009; Adesse et al., 2011a), making these compounds potential therapeutic candidates for treatment of Chagas disease.

Besides their involvement in the early steps of *T. cruzi*–cardiomyocyte recognition, ECM components also present a striking role in chagasic cardiomyopathy pathogenesis since their accumulation leads to fibrosis, disposing patients to heart failure and ventricular arrhythmias (Rassi et al., 2010, 2012). In experimental systems, ECM accumulation begins during the late acute phase of infection (Andrade et al., 1989; Calvet et al., 2004), concomitantly with the onset of inflammatory infiltrates, indicating that the process of fibrogenesis is triggered in the early stages of *T. cruzi* infection. A general increase in ECM transcripts and expression was detected by microarray analysis in acute infection (Garg et al., 2003). Cardiac hypertrophy and ECM remodeling were also seen in a *T. cruzi*-infected 3D cardiomyocyte model (Garzoni et al., 2008; **Figure 2**). Surprisingly, reduction of ECM in *T. cruzi*-infected cardiomyocytes was detected by silver staining in acute infection in mice (Factor et al., 1993). Additionally, *T. cruzi*-mediated down-regulated ECM gene expression in cardiomyocyte cultures (Goldenberg et al., 2009; Manque et al., 2011) and a reduction in the synthesis and spatial distribution of fibronectin were detected in heavily infected cardiomyocytes (Calvet et al., 2004; **Figure 2**) even after TGF-β stimulation (Calvet et al., 2009), suggesting that despite the general enhancement of ECM in the heart, the cells harboring the parasites display low ECM expression. The anti-fibrogenic effect of *T. cruzi* has also been seen in human dermal fibroblasts, with repression of transcription factors that regulate expression of fibroblast genes involved in wound repair and tissue remodeling, including ctgf/ccn2 connective tissue growth factor gene, followed by down-regulation of ECM proteins such as fibronectin and collagen I, suggesting another route of parasite dissemination and infection (Unnikrishnan and Burleigh, 2004; Mott et al., 2011).

Another point worth discussing relates to the ability of *T. cruzi* to modulate host cell apoptosis, or programed cell death, a physiological process of cell replacement to maintain tissue homeostasis (Mondello and Scovassi, 2010). Pathogens can hijack the host cell apoptotic machinery as an offensive strategy to eliminate the host’s immune response (Lamkanfi and Dixit, 2010). Both anti- and pro-apoptotic gene expression are differentially modulated during *T. cruzi*–cardiomyocyte infection, leading to a balance between cell death and survival at different stages of infection (Manque et al., 2011). Induction of apoptosis by *T. cruzi* infection is controversial and seems to be dependent on host cell and parasite genotype (de Souza et al., 2003;

Aoki et al., 2006; Petersen et al., 2006). While fibroblasts are refractory to apoptosis, cardiomyocytes and macrophages differentially undergo apoptosis after *T. cruzi* infection, the latter cell type being highly susceptible. Still, cardiomyocytes infected with *T. cruzi* clone Dm28c have higher levels of apoptosis compared to infection with strains Y and CL (de Souza et al., 2003). Furthermore, the intracellular parasites themselves also undergo apoptosis, hinting at a host attempt to control parasite burden (de Souza et al., 2003, 2010). Interestingly, it has been shown that α2-macroglobulin, a plasma proteinase inhibitor, regulates apoptosis in *T. cruzi*-infected cardiomyocytes and macrophages, impairing the cell death process (de Souza et al., 2008). In contrast, an anti-apoptotic effect has also been demonstrated in cardiac cells (Petersen et al., 2006). The prevention of apoptosis appears to be related to NF-κB activation by inhibiting the signaling of caspases, thus avoiding cell death. Thus, avoidance of apoptosis reduces cardiac damage and may be responsible for the persistence of *T. cruzi* infection.

While our knowledge of *T. cruzi*–host cell interactions has greatly improved, many questions remain open. There are still gaps in our understanding of the molecular interactions involved in cellular recognition and/or signaling pathway in most of the mechanisms of invasion. What are the critical links between these processes? And little is still known about the cooperative role played by the host cell in parasite intracellular growth and differentiation. These questions demand deeper investigation.

## Conflict of Interest Statement

The authors declare that the research was conducted in the absence of any commercial or financial relationships that could be construed as a potential conflict of interest
